# Supplementary material for: Benzamide Derivatives Targeting the Cell Division Protein FtsZ: Modifications of the Linker and the Benzodioxane Scaffold and Their Effects on Antimicrobial Activity
Source: Antibiotics (Basel). 2020 Apr 4;9(4):160. doi: 10.3390/antibiotics9040160 (PMC7235863; doi:10.3390/antibiotics9040160)
Supplement: Supplementary File 1 [file antibiotics-09-00160-s001.zip › SupportingInformation_Antibiotics_2020.docx]

Supporting Information

**“Benzamide derivatives targeting the cell division protein FtsZ: modifications on the linker and on the benzodioxane scaffold and their effect on antimicrobial activity”**

**Valentina Straniero *, Lorenzo Suigo, Andrea Casiraghi, Victor Sebastián-Pérez, Martina Hrast, Carlo Zanotto, Irena Zdovc, Carlo De Giuli Morghen, Antonia Radaelli and Ermanno Valoti ***

Sommario

[**3-(1,4-benzoxathian-2-yl)-2,6-difluorobenzamide (1)** 2](#_Toc32330905)

[**3-(1,4-benzoxathiane-4,4-dioxide-2-yl)-2,6-difluorobenzamide** **(2)** 3](#_Toc32330906)

[**3-(1,4-benzoxathian-3-yl)-2,6-difluorobenzamide (3)** 4](#_Toc32330907)

[**3-(1,4-benzoxathiane-4,4-dioxide-3-yl)-2,6-difluorobenzamide** **(4)** 5](#_Toc32330908)

[**3-(1,4-benzodithianyl)methoxy-2,6-difluorobenzamide (5)** 6](#_Toc32330909)

[**3-(1,4-benzoxathian-2-yl)ethoxy-2,6-difluorobenzamide (6)** 7](#_Toc32330910)

[**2,6-Difluoro-3-(1,4-benzodioxan-2-yl)ethoxybenzamide (7)** 8](#_Toc32330911)

[**2,6-Difluoro-3-(1,4-benzodioxan-2-yl)propoxy)benzamide (8)** 9](#_Toc32330912)

## **3-(1,4-benzoxathian-2-yl)-2,6-difluorobenzamide (1)**

# **3-(1,4-benzoxathiane-4,4-dioxide-2-yl)-2,6-difluorobenzamide** **(2)**

**3-(1,4-benzoxathian-3-yl)-2,6-difluorobenzamide (3)**

# **3-(1,4-benzoxathiane-4,4-dioxide-3-yl)-2,6-difluorobenzamide** **(4)**

# **3-(1,4-benzodithianyl)methoxy-2,6-difluorobenzamide (5)**

# **3-(1,4-benzoxathian-2-yl)ethoxy-2,6-difluorobenzamide (6)**

# **2,6-Difluoro-3-(1,4-benzodioxan-2-yl)ethoxybenzamide (7)**

# **2,6-Difluoro-3-(1,4-benzodioxan-2-yl)propoxy)benzamide (8)**
